# In search of the RNA world on Mars

**DOI:** 10.1111/gbi.12433

**Published:** 2021-02-10

**Authors:** Angel Mojarro, Lin Jin, Jack W. Szostak, James W. Head, Maria T. Zuber

**Affiliations:** ^1^ Department of Earth, Atmospheric and Planetary Sciences Massachusetts Institute of Technology Cambridge MA USA; ^2^ Department of Molecular Biology, and Center for Computational and Integrative Biology Massachusetts General Hospital Boston MA USA; ^3^ Department of Earth, Environmental and Planetary Sciences Brown University Providence RI USA

**Keywords:** Mars, origins of life, RNA world

## Abstract

Advances in origins of life research and prebiotic chemistry suggest that life as we know it may have emerged from an earlier RNA World. However, it has been difficult to reconcile the conditions used in laboratory experiments with real‐world geochemical environments that may have existed on the early Earth and hosted the origin(s) of life. This challenge is due to geologic resurfacing and recycling that have erased the overwhelming majority of the Earth's prebiotic history. We therefore propose that Mars, a planet frozen in time, comprised of many surfaces that have remained relatively unchanged since their formation > 4 Gya, is the best alternative to search for environments consistent with geochemical requirements imposed by the RNA world. In this study, we synthesize in situ and orbital observations of Mars and modeling of its early atmosphere into solutions containing a range of pHs and concentrations of prebiotically relevant metals (Fe^2+^, Mg^2+^, and Mn^2+^) spanning various candidate aqueous environments. We then experimentally determine RNA degradation kinetics due to metal‐catalyzed hydrolysis (cleavage) and evaluate whether early Mars could have been permissive toward the accumulation of long‐lived RNA polymers. Our results indicate that a Mg^2+^‐rich basalt sourcing metals to a slightly acidic (pH 5.4) environment mediates the slowest rates of RNA cleavage, though geologic evidence and basalt weathering models suggest aquifers on Mars would be near neutral (pH ~ 7). Moreover, the early onset of oxidizing conditions on Mars has major consequences regarding the availability of oxygen‐sensitive metals (i.e., Fe^2+^ and Mn^2+^) due to increased RNA degradation rates and precipitation. Overall, (a) low pH decreases RNA cleavage at high metal concentrations; (b) acidic to neutral pH environments with Fe^2+^ or Mn^2+^ cleave more RNA than Mg^2+^; and (c) alkaline environments with Mg^2+^ dramatically cleaves more RNA while precipitates were observed for Fe^2+^ and Mn^2+^.

## 1 INTRODUCTION

The origins of life can best be understood as a series of plausible steps culminating in the emergence of a “self‐sustaining chemical system capable of Darwinian evolution” (NASA Astrobiology). However, life as we know it is a highly complex collection of molecular machinery and genetic information. The central dogma of molecular biology stipulates that deoxyribonucleic acid (DNA) makes ribonucleic acid (RNA) via transcription, RNA makes protein via translation, and information cannot be transferred backwards from proteins to nucleic acids (Crick, 1970). Fundamentally, neither DNA, RNA, nor proteins can exist without the others as they do today. Nevertheless, this dilemma belies the fact that the capability of translating information between dissimilar polymers (e.g., polynucleotides to polypeptides) is mediated by the ribosome, an RNA enzyme (Cech, 2000). This is significant because the ribosome is arguably an evolutionary anachronism from a period where RNA polymers acted as both enzymes (protein) and information storage (DNA) (Petrov et al., 2015). Additional discoveries of structural and regulatory RNA molecules (Breaker, 2012) suggest that life may have emerged from an earlier RNA world dominated by ribozymes (e.g., the ribosome) (Gilbert, 1986) and ribonucleotide‐containing molecules (e.g., adenosine triphosphate—ATP) (Hernández‐Morales et al., 2019) catalyzing reactions and mediating a protometabolism.

Under the RNA world scenario, abiotic synthesis of simple RNA molecules from common molecular feedstocks in geologically relevant environments (e.g., Patel et al., 2015) would have given rise to self‐assembling protocellular systems (Joyce & Szostak, 2018). Thereafter, increasingly complex RNA polymers capable of both hereditary storage and autocatalysis would precede the DNA–RNA–protein world (Bernhardt, 2012). To test the RNA world hypothesis, investigators have experimentally demonstrated the following: (a) abiotic RNA synthesis (e.g., Powner et al., 2009); (b) non‐enzymatic RNA replication (Adamala & Szostak, 2013; Jin et al., 2018); (c) directed evolution and fitness landscapes yielding persistent RNA motifs (Jimenez et al., 2013) and functional ribozymes (Voytek & Joyce, 2007); and (d) the co‐synthesis of RNA, amino acids, and lipids (Patel et al., 2015).

Although strides in prebiotic chemistry have demonstrated the viability of an origin of life via the RNA world, a long‐standing criticism is that RNA is inherently unstable due to the presence of a nucleophilic 2’‐hydroxyl group which readily catalyzes cleavage of the 5’,3’‐phosphodiester bond (Li & Breaker, 1999). Because of this characteristic, RNA is deemed an ephemeral molecule that is unlikely to accumulate, functionalize, and precipitate life in a prebiotic world. Researchers have therefore directed efforts toward determining particular conditions or cofactors which can stabilize RNA in real‐world environments. Experimental work suggests the following: (a) RNA is most chemically stable between pH 4 – 5 (Bernhardt & Tate, 2012; Oivanen et al., 1998) and near 0°C (Kua & Bada, 2011; Levy & Miller, 1998); (b) metal cofactors such as Fe^2+^, Mg^2+^, and Mn^2+^ facilitate the folding of RNA polymers into stable secondary and tertiary structures (Bowman et al., 2012; Laing et al., 1994; Petrov et al., 2012); (c) copolymers such as polypeptides and polysaccharides can favor specific polynucleotide conformations, resulting in persistent structures and vice versa (Frenkel‐Pinter et al., 2020; Runnels et al., 2018); (d) mutually stabilizing peptide‐RNA conformations rapidly denature above 45°C (Frenkel‐Pinter et al., 2020); and (e) folding of many RNA sequences decreases rapidly above 30°C (Moulton et al., 2000).

Still, it has been difficult to confidently determine the dynamic environments that could have existed on the Hadean Earth and hosted the origin of life. This challenge is in part due to geologic resurfacing and recycling that have erased the overwhelming majority of the Earth's prebiotic history (Marchi et al., 2014). Nevertheless, we can speculate that the likeliest time interval for the origin of life on Earth can be constrained by the accretion of the first continents following a Moon‐forming impact (Monteux et al., 2016) or possible late veneer impactor (Genda et al., 2017) ~4.5–4.4 Gya and the earliest unambiguous biological structures at ~ 3.5 Gya (Allwood et al., ,,,^2006^, ^2009^). Given this consideration, the best alternative is to search for environments consistent with RNA stability on Mars, a planet frozen in time, preserving primordial surfaces which have remained relatively unchanged since they formed > 4 Gya (Hartmann & Neukum, 2001). Perhaps a window into the Hadean on Earth, the Noachian on Mars is characterized by meteoritic bombardment and punctuated aqueous activity resulting in extensive groundwater circulation (Ehlmann et al., 2011), valley networks (Fassett & Head, 2011), and long‐lived lacustrine environments (Goudge et al., 2012, 2015; Grotzinger et al. 2014). Some researchers would argue that life began on Mars and was transported to the Earth around the timing of the earliest putative biosignatures ~ 3.8 Gya found in metasedimentary rocks (Alleon & Summons, 2019; Benner & Kim, 2015; Hassenkam et al., 2017; Tashiro et al., 2017). That is, non‐sterilizing lithological exchange between Mars and Earth from impact ejecta produced during the presumed Late‐Heavy Bombardment period (Boehnke & Harrison, 2016; Gomes et al., 2005) may have transported viable microbes between planets resulting in ancestrally related life (Gladman et al., 1996; Weiss, 2000).

The case for an origin of life on Mars relies on prebiotic environments that are inferred to be analogous to early environments on Earth (Sasselov et al., 2020), common molecular feedstocks (including cometary sources) (Callahan et al., 2011), and plausible reactive pathways predicted on Earth that are applicable on Mars (e.g., Ritson et al., 2018) which may have resulted in parallel events in accordance with the RNA world hypothesis (Benner & Kim, 2015). This notion is further supported by in situ detection of boron (Gasda et al., 2017), which is considered crucial to stabilize ribose in the formose reaction (Furukawa & Kakegawa, 2017), experimental work that predicts higher phosphate bioavailability on Mars (Adcock et al., 2013), and the detection of clays (Ehlmann et al., 2011) that have been demonstrated to assist in non‐enzymatic RNA polymerization (Ferris, 2006). This Mars origin of life hypothesis suggests that past or present Martian life may have utilized known building blocks (e.g., nucleic acids, sugars, amino acids) and closely resembled life as we know it. Moreover, if life exists on Mars today, it could theoretically be detected by means of nucleic acid (DNA and RNA) sequencing (Carr et al. 2017; Mojarro et al., 2019). Assuming that viable RNA was being delivered to Mars via unspecified sources (e.g., cometary or in situ synthesis) to UV‐shielded aqueous environments (Cockell et al., 2000), here we investigate whether early Mars was permissive toward the accumulation of long‐lived RNA polymers. We anticipate our findings could provide insight into potential mechanisms, environments, and requirements necessary for sustaining an RNA world on the early Earth.

## 2 MATERIALS AND METHODS

### 2.1 Approach

The surface of Mars displays evidence for alternating climate regimes at regional‐to‐global magnitudes that have evolved on variable time scales not dissimilar to the Earth (McLennan et al., 2019). In general, early Mars contained a broad range of geochemical environments (e.g., acidic to alkaline) primarily influenced by redox chemistry. In this study, we synthesize in situ and orbital observations and modeling of the early Martian atmosphere in order to extrapolate representative solutions containing a range of pHs and metals analogous to various candidate aqueous environments on Mars. Below we detail our experimental design, which involves incubating a hybrid RNA–DNA oligomer (simply referred to as the RNA‐containing oligomer) to quantify the hydrolysis rate of the 5’,3’‐phosphodiester bond at a single ribonucleotide within the aforementioned solutions. The goal of this study is to understand the influence of bedrock composition (e.g., mafic‐ultramafic, iron‐rich, and magnesium‐rich) and subsequent weathering of prebiotically relevant metals (i.e., Fe^2+^, Mg^2+^, and Mn^2+^) which have been demonstrated to catalyze hydrolysis (Fedor, 2002), folding (Laing et al., 1994), non‐enzymatic replication (Adamala & Szostak, 2013), translation (Bray et al., 2018), and impart novel catalytic function (Hsiao et al., 2013) on RNA stability. Furthermore, pH is simultaneously adjusted to reflect the composition of a hypothetical anoxic and CO_2_‐dominated atmosphere at variable pressures in equilibrium with surface waters and pHs found at an average acid vent and an average alkaline vent (Kua & Bada, 2011). The end result is an analysis of single‐stranded RNA stability and degradation kinetics in an array of simulated prebiotic geochemical spaces on Mars.

### 2.2 RNA‐containing oligomer

**Figure 1:**
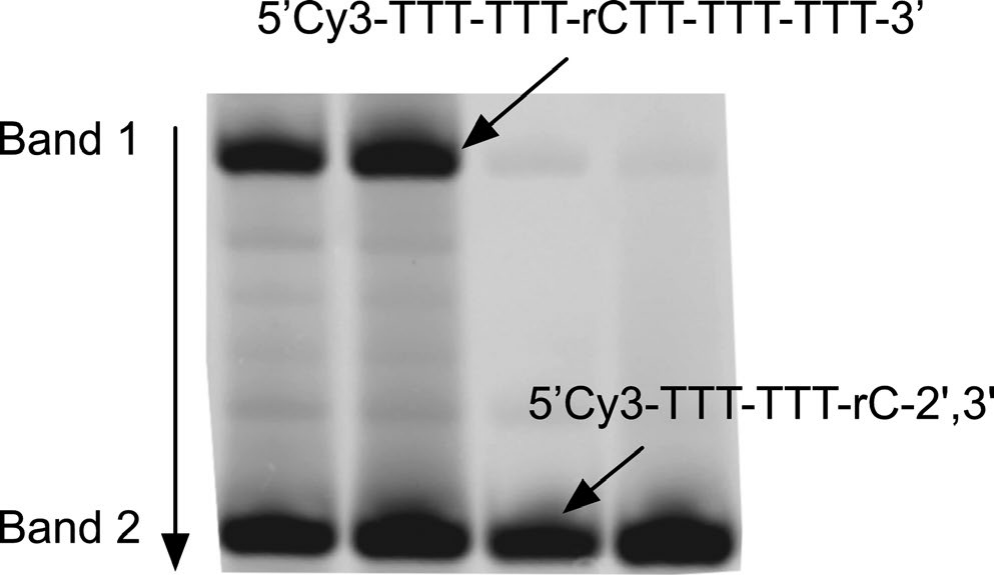
RNA‐containing oligonucleotide cleavage assay. A hybrid RNA–DNA oligomer, 5’‐Cy3‐TTT‐TTTrCTT‐TTT‐TTT‐3’, was designed to contain a single ribonucleotide (r) in between a chain of deoxyribonucleotides which could allow us to quantify cleavage at a single site. Representative gel scan displays two fluorescent bands belonging to either the intact 15‐mer (Band 1) or the residual 7‐mer (Band 2) cleaved at the single ribonucleotide site

A fluorescently labeled hybrid RNA‐DNA oligomer, 5’‐Cy3‐TTT‐TTT‐rCTT‐TTT‐TTT‐3’, was designed to contain one ribonucleotide (r) in between deoxyribonucleotides, allowing us to quantify hydrolysis at a single cleavage site by transesterification on a urea polyacrylamide gel (Figure 1) (e.g., Adamala & Szostak, 2013). This characteristic is important because it limits cleavage products to either: 1) an intact 15‐mer (5’‐Cy3‐TTT‐TTT‐rCTT‐TTT‐TTT‐3’—band 1) or 2) a 2’‐3’ cyclic phosphate terminated 7‐mer (5’‐Cy3‐TTT‐TTT‐rC‐2’,3’—band 2) plus a second (non‐fluorescent) 8‐mer product that begins with a 5’‐hydroxyl (5’‐TT‐TTT‐TTT‐3’) (Figure 1). An oligomer solely containing ribonucleotides would otherwise appear as a smear (on a gel) due to cleavage at any 5’,3’‐phosphodiester linkage resulting in a distribution of product sizes (e.g., 15‐mer, 14‐mer, and 13‐mer). RNAse‐free and HPLC‐purified oligos were ordered from Integrated DNA Technologies (Custom RNA oligos).

### 2.3 Relevant observations—Hadean Earth and the last universal common ancestor (LUCA)

Little‐to‐no record exists of Earth's prebiotic history during the Hadean. Analysis of isotopic signatures and inclusions preserved within zircons (e.g., the oldest‐surviving crustal material) suggest that a global ocean (Mojzsis et al., 2001) and the first continents (Wilde et al., 2001) existed at this time. Furthermore, analysis of ~ 3.8 Gya metasedimentary rocks perhaps indicates that life may have also originated within the Hadean (Alleon & Summons, 2019; Hassenkam et al., 2017; Tashiro et al., 2017). Given this dearth of information, deep‐sea hydrothermal vents have often been invoked as a likely setting for the origins of life on Earth (e.g., Martin et al., 2008) due to: (a) their predicted availability on the early Earth providing (b) a steady source CO_2_ and H_2_ (i.e., derived by serpentinization) that could possibly undergo Fischer‐Tropsch synthesis and reductive transformation into complex biomolecules and (c) sustain thermophilic methanogens/acetogens via the acetyl‐CoA pathway.

Phylogenetic reconstructions of the last universal common ancestor (LUCA), however, have since indicated that LUCA was most likely a mesophilic surface‐dweller capable of UV repair (e.g., Cantine & Fournier, 2018). Ribosomal RNA (rRNA) ancestral state reconstructions show a GC‐content consistent with mesophilic optimal growth temperatures (Galtier, 1999; Groussin et al., 2013) while protein reconstructions demonstrate a depletion of thermostable amino acids (Boussau et al., 2008; Zeldovich et al., 2007) and a divergence between informational and metabolic families inconsistent with a thermophilic origin (Berkemer & McGlynn, 2020). In addition, from a building block perspective, prior work by Kua & Bada, 2011 has demonstrated that ribose, cytosine (i.e., the least stable nucleobase), and the phosphodiester linkage are most stable at 0º C while Levy & Miller, 1998 concluded a high‐temperature origin could not involve the canonical genetic code. Altogether, these results indicate thermophily may have been an evolutionary adaptation in response to a thermophilic bottleneck (e.g., the late‐heavy bombardment) (Boussau et al., 2008).

Overall, phylogenetics and experimental work on RNA synthesis, stability, and function strongly indicates a planetary surface origin driven by UV photochemistry (Patel et al., 2015), common molecular feedstocks (e.g., HCN) (Parkos et al., 2018; Patel et al., 2015; Toner & Catling, 2019), cool temperatures (Frenkel‐Pinter et al., 2020; Kua & Bada, 2011; Levy & Miller, 1998; Moulton et al., 2000), and wet‐dry cycling (Sasselov et al., 2020). Therefore, while the Hadean prebiotic record has been lost, we may be able to elucidate candidate environments by studying the surface of Mars.

### 2.4 Relevant observations—Mars

**Figure 2:**
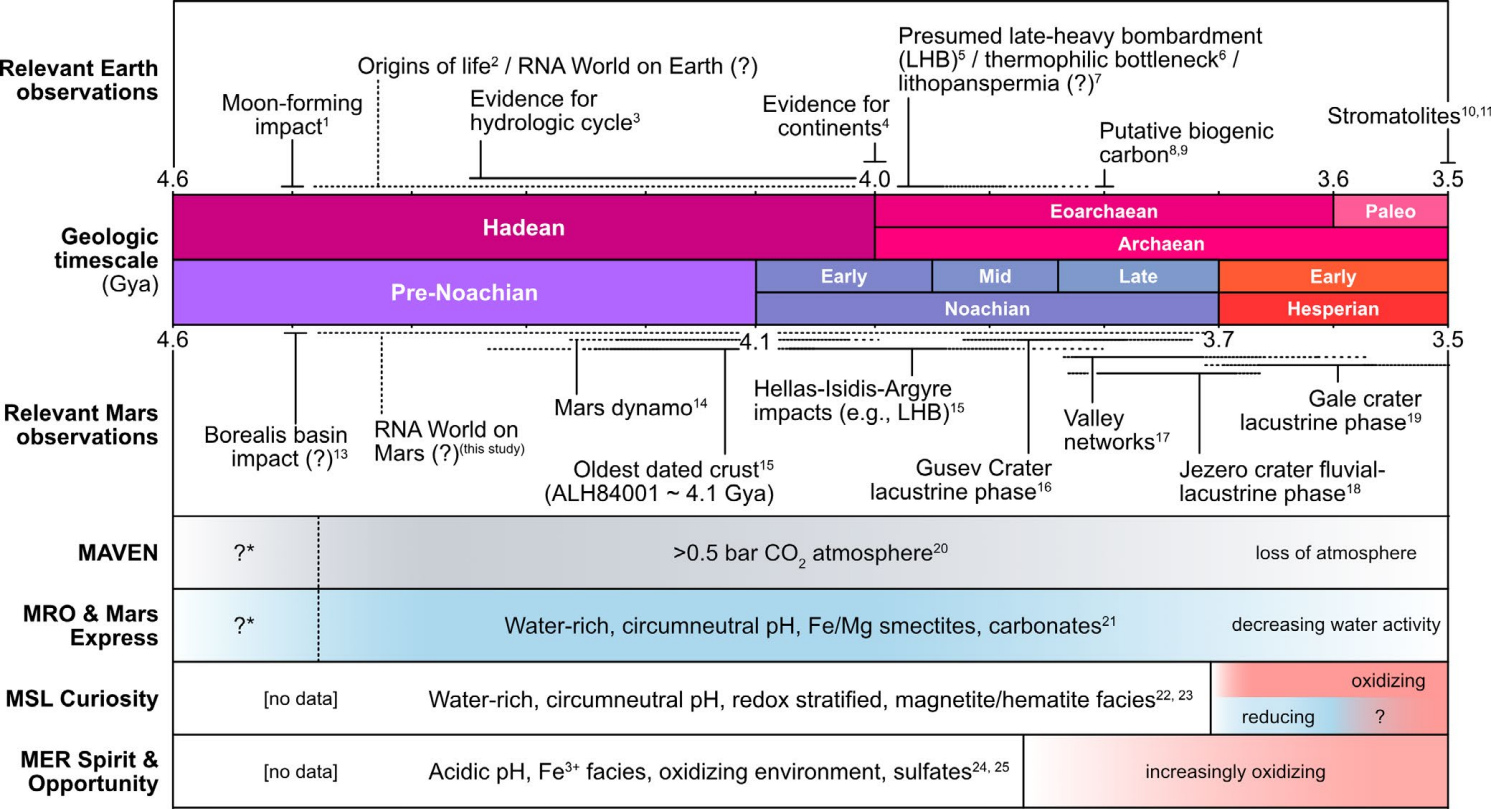
Relevant observations of Earth and Mars. Geologic resurfacing and recycling have erased the overwhelming majority of the Earth's prebiotic history during the Hadean when life may have originated. We propose that Mars, a planet comprised of surfaces that have remained relatively unchanged since their formation, is the best alternative to search for environments consistent with geochemical requirements imposed by the RNA world. *uncertain timing, ^1^Monteux et al. 2016, ^2^Pearce et al. 2018, ^3^Mojzsis et al. 2001, ^4^Wilde et al. 2001, ^5^Gomes et al. 2005, ^6^Boehnke & Harrison, 2016, ^7^Boussau et al. 2008, ^8^Gladman et al. 1996, ^9^Hassenkam et al. 2017, ^10^Tashiro et al. 2017, ^11^Allwood et al. 2006, ^12^Allwood et al. 2009, ^13^Andrews‐Hanna et al. 2010, Lillis et al. 2013, ^15^Fassett & Head, 2011, ^16^Cabrol et al. 2003, ^17^Fassett & Head, 2008a, ^18^Goudge et al. 2018, ^19^Grotzinger et al. 2014, ^20^Jakosky et al. 2017, ^21^Ehlmann & Edwards, 2014, ^22^Grotzinger et al. 2014, ^23^Hurowitz et al. 2017, ^24^Squyres et al. 2006, ^25^Ming et al. 2008

Below we list relevant observations from rover/orbiter mission and modeling that have been utilized to synthesize candidate aqueous environments and are relevant for our discussion on the viability of an RNA world on Mars (Figure 2).

#### 2.4.1 Mars Exploration Rover (MER) Opportunity

Sedimentary rocks exposed in the Meridiani Planum region (Burns formation) of Mars record alternating periods of acidic groundwater flow (pH ~ 2–4) and desiccation under highly oxidizing conditions (Klingelhofer, 2004; McLennan, 2012; Squyres & Knoll, 2005; Squyres et al., 2006).

#### 2.4.2 Mars Exploration Rover (MER) Spirit

Widespread Fe^3+^‐sulfate soils (e.g., jarosite) at Gusev Crater indicate acid weathering of primarily olivine‐rich outcrops in a possible hydrothermal environment (Ming et al., 2008; Yen et al., 2008).

#### 2.4.3 Mars Science Laboratory (MSL) Curiosity Rover

Sedimentary rocks analyzed at Gale Crater (Bradbury‐Mount Sharp groups) reflect a long‐lived lacustrine environment with circumneutral pH waters and variable redox states. This is indicated by the presence of manganese oxide (Mn^2+^) deposits, magnetite‐silica facies (Fe^2+^), and hematite‐phyllosilicate facies (Fe^3+^) (Grotzinger et al., 2014, 2015; Hurowitz et al., 2017; Lanza et al., 2016).

#### 2.4.4 Mars Reconnaissance Orbiter (MRO) and Mars Express Orbiter

Crustal Fe‐Mg smectites indicate global groundwater circulation (Ehlmann et al., 2011) consistent with work, suggesting subsurface waters were primarily anoxic, Fe^2+^‐rich, and circumneutral pH which became rapidly acidified due to atmospheric O_2_ or photo‐oxidation of Fe^2+^ to Fe^3+^ at the Martian surface (Hurowitz et al., 2010). Mg^2+^‐rich carbonate deposits near Nili Fossae indicate neutral to alkaline pH waters likely in contact with a CO_2_ atmosphere (Ehlmann et al., 2008). In addition, global imaging of surface features has revealed and abundant suite of aqueous environments that include fluvial valley networks (e.g., Fassett & Head, 2008; Hynek et al., 2010), playas (e.g., Andrews‐Hanna et al., 2010), open‐basin lakes (e.g., Fassett & Head, 2008b; Goudge et al., 2012), and closed‐basin (endorheic) lakes (e.g., Goudge et al., 2015).

#### 2.4.5 Mars Atmosphere and Volatile Evolution (MAVEN) Orbiter

Isotopic evidence indicates a continuous loss of a ≥ 0.5 bar CO_2_‐dominated atmosphere (Jakosky et al., 2017) since the early Noachian ~ 4.1 Gya due to erosion by solar wind when the Martian dynamo is thought to have shut down (Lillis et al. 2013).

#### 2.4.6 Atmospheric Modeling

Various models have suggested a range of atmospheric compositions (e.g., H_2_, CO_2_, H_2_O, SO_2_, H_2_S); however, many have not been able to resolve counteracting cooling effects of atmospheric density and albedo due to aerosol and cloud formation (e.g., Palumbo et al., 2018; Tian et al., 2010). Primarily due to lower solar luminosity values in early history, atmospheric general circulation models (e.g., Forget et al., 2013; Palumbo & Head, 2018; Wordsworth et al., 2013) have found it difficult to maintain the > 273 K mean annual temperature (MAT) seemingly required to support a “warm and wet” or “warm and arid” early Mars and valley network formation (e.g., Craddock et al., 2003). Most recently, work by Ramirez et al. 2020 has proposed that CO_2_‐H_2_ collision‐induced absorption could have raised mean surface temperatures above 273 K. That is, assuming the presence of a hypothetical northern lowland ocean (Chan et al., 2018), atmospheric pressures as low as 0.55 bar CO_2_ and 1% H_2_ may have sustained a relatively warm and wet early Mars (Ramirez, 2017). Instead, these models suggest a “cold and icy” early Mars climate (Head & Marchant, 2014) in which snow and ice were deposited in the uplands, and episodic transient heating events caused melting and runoff to form the valley networks and lakes. Among the candidate transient events proposed are those due to: (a) spin‐axis/orbital variations influencing peak annual and seasonal temperatures (Palumbo et al., 2018); (b) volcanic eruptions (e.g., Halevy & Head, 2014); (c) impact events (e.g., Palumbo & Head, 2018; Segura et al., 2008; Steakley et al., 2019; Turbet et al., 2020); (d) subsurface radiolytic H_2_ production and release (e.g., Tarnas et al., 2018); and (e) collision‐induced absorption temperature amplifications during transient CO_2_ and methane release events (e.g., Wordsworth et al., 2017).

### 2.5 Mars prebiotic geochemical solutions

Given the aforementioned observations (Figure 2), we designed our experiments to simulate environments that are in equilibrium with an anoxic CO_2_‐dominated atmosphere at variable pressures (10 bar, 0.1 bar, »0.1 bar) inducing their respective shifts in pH (5.4, 6.7, 8) as calculated by Kua & Bada, 2011. Solutions representing an average acidic vent at pH 3.2 and average alkaline vent at pH 9 were also included. Each pH solution contained 0, 2.5, 5, 10, 25, and 50 mM of Fe^2+^, Mg^2+^, or Mn^2+^ intended to represent a range of dissolved metal concentrations within the water column, variable weathering, residence times, and wet‐dry cycling. High values additionally allow us to easily measure kinetics and extrapolate rates of catalysis for more geochemically reasonable metal concentrations. Mixtures of Fe^2+^ and Mg^2+^ at 50:50, 20:80, and 80:20 (25 mM total) were included to represent metal concentrations derived from variable bedrock compositions; in particular, 20:80 Fe^2+^:Mg^2+^ is closest to the average crustal composition on Mars (Mittlefehldt, 1994). A total of 5 pH conditions, 3 metals at 5 concentrations, 3 basalt analogs, and 5 negative controls resulted in 95 unique conditions.

Samples were prepared by mixing stock buffer solutions of 1 M Glycine‐HCL (Sigma‐Aldrich, 50,046) at pH 3.2, 0.5 M MES (Sigma‐Aldrich, 76,039) at pH 5.4 and 6.7, 1 M Tris pH 8 (Thermo Fisher, AM9849), and 1 M Tris pH 9 (Millipore, 9295‐OP) with stock solutions of 0.5 M ammonium iron(II) sulfate hexahydrate (Sigma‐Aldrich, 09,719), 0.5 M manganese(II) chloride tetrahydrate (Sigma‐Aldrich, 63,535), or 0.5 M magnesium chloride (Thermo Fisher, AM9530G). A typical RNA incubation consisted of 250 mM buffer, 1 mM EDTA (Thermo Fisher, AM9260G), 0–50 mM of metals, and 5 µM of the RNA oligomer in a final 20 µl reaction successively added (in that order) into a 0.2‐mL PCR tube (Thermo Fisher, AM12230). In particular, 1mM EDTA added prior to the addition of Fe^2+^, Mg^2+^, or Mn^2+^ was used to bind trace levels of transition metals that might contaminate solutions. All stock solutions were sparged with argon and stored inside an anaerobic glove box (Coy). The atmosphere inside the glove box was N_2_ with 2.5% – 3% H_2_ and internal circulation through a platinum catalyst maintained residual oxygen levels below 10 ppm. All RNA reactions occurred inside the glove box on a miniPCR mini16 thermocycler (Amplyus, QP‐1016‐01) kept at 75°C in order to facilitate rapid RNA degradation.

### 2.6 RNA degradation quantification

All RNA degradation experiments were quantified via urea polyacrylamide gel electrophoresis (National Diagnostics, EC‐830 & EC‐840) followed by imaging on a Typhoon 9,410 (GE Healthcare). Two fluorescent bands were detected representing either the intact 15‐mer (5’‐Cy3‐TTT‐TTT‐rCTT‐TTT‐TTT‐3’—band 1) or the residual 7‐mer (5’‐Cy3‐TTT‐TTT‐rC‐2’,3’—band 2) cleaved at the single ribonucleotide site (Figure 1). Gel images were then analyzed with ImageQuant TL 7.1 (GE Healthcare) to determine the percent intact oligo (i.e., p_t_ = band 1/ band 1 + band 2) at t = time.

### 2.7 RNA degradation kinetics

**Figure 3:**
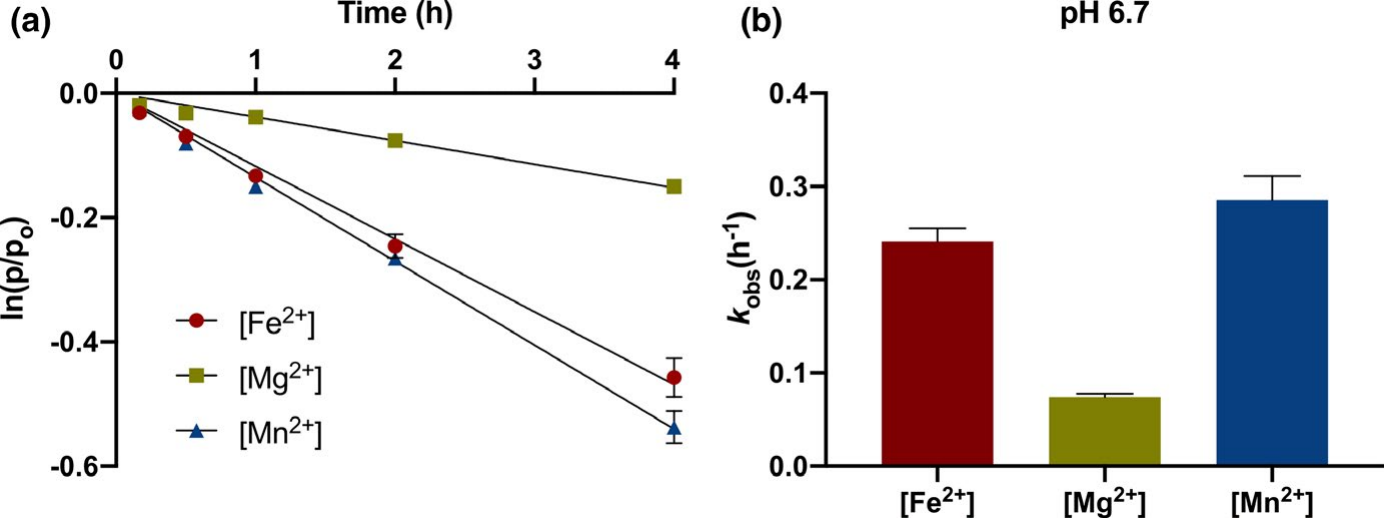
Metal ion catalysis of RNA degradation. Plot of oligonucleotide strand cleavage at the single ribonucleotide site as measured by urea polyacrylamide gel electrophoresis with 50 mM Fe2+ (●), 50 mM Mg2+ (■), and 50 mM Mn2+ (▲) at pH 6.7. (a) The natural logarithm of the fraction of un‐cleaved (e.g., intact) RNA‐containing oligomer with time (h) was fit to a linear regression ln[pt] = ‐kt + ln[po] and (b) the slope yielded our pseudofirst‐order rate constants (kobs(h‐1). [Fe2+]: ln(p/po) = −0.117h, R2 = 0.997; [Mg2+]: ln(p/po) = −0.038h, R2 = 0.989; [Mn2+]: ln(p/po) = −0.135h, R2 = 0.999

**Figure 5:**
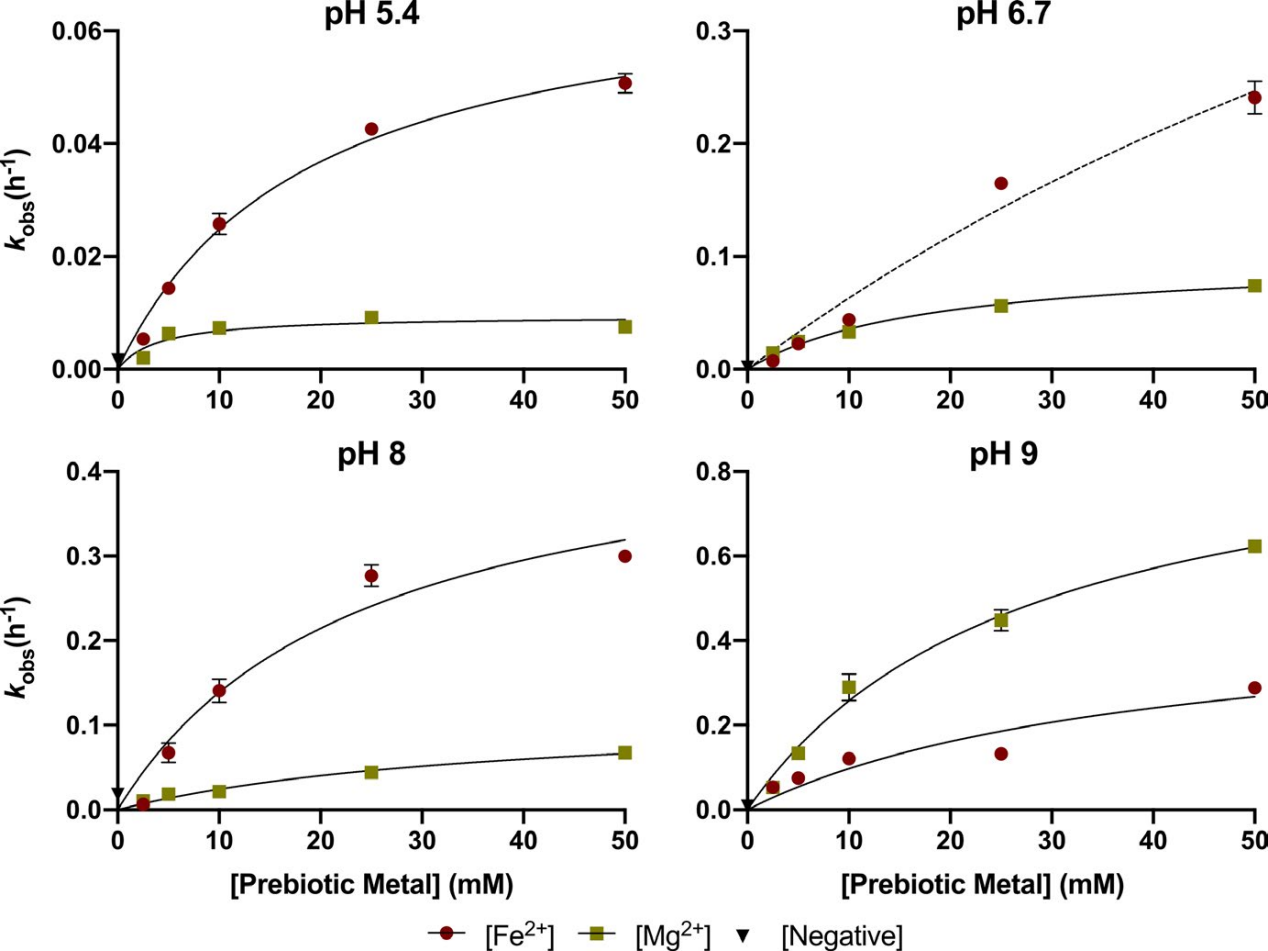
Characterization of RNA degradation kinetics. Saturation curves of Fe2 + and Mg2+ cleavage fitted to the one‐site binding model. Within the range tested, we observe greater RNA stability at pH 5.4 where the maximum rate of catalysis (Bmax) with Mg2+ is lower than with Fe2+. Experiments with Fe2+ at pH 6.7 did not reach saturation. Error bars represent SEM (*n* = 2)

Of 5 µM of the RNA‐containing oligomer was incubated in each of the Mars solutions as described above at 75 ºC (*n* = 2) inside an anaerobic chamber. 1 µl aliquot time points were taken at 5, 15, 30, 60, and 120 min for pH 6.7, 8, and 9 and at 10, 30, 60, 120, and 240 min for pH 3.2 and 5.4. Aliquots were added to 25 µl of a kill buffer solution of 8 M urea, 1x TBE (Thermo Fisher, 15,581,044), and 100 mM EDTA in order to arrest further catalysis and collect representative time point measurements. Specifically, EDTA–metal complexes in aliquot‐kill buffer solutions are not active because EDTA sequesters (i.e., chelates) metal ions and keeps them away from the substrate (i.e., RNA‐containing oligomer). Samples were then removed from the anaerobic chamber, and 5 µl of the kill buffer‐time point mixture (2 picomoles RNA) was taken for gel electrophoresis and quantification. Pseudofirst‐order reaction kinetics *k*_obs_(h^‐1^) of RNA hydrolysis were determined with the following relationship, ln[p_t_] = ‐*k*t + ln[p_o_] where p_t_ = percent intact oligo at t = time n and p_o_ = percent intact oligo at t = 0 (i.e., 100%) (Figure 3). We then fit *k*_obs_(h^‐1^) versus metal concentration (mM) to a one‐site binding model by assuming that hydrolysis is preceded by metal binding at the single ribonucleotide (e.g., one binding site per RNA‐containing oligomer). This model is represented by *k*_obs_(h^‐1^) = B_max_(h^‐1^) * metal / (K_d_ + metal) where B_max_ = maximum metal binding (i.e., resulting in maximum rate of cleavage) and K_d_ = the metal concentration required to achieve half the maximum metal‐ribonucleotide binding (and ensuing cleavage) (Figure 5). These relationships subsequently allow us to extrapolate hydrolysis rates to lower/higher metal concentrations.

## 3 RESULTS

**Figure 4:**
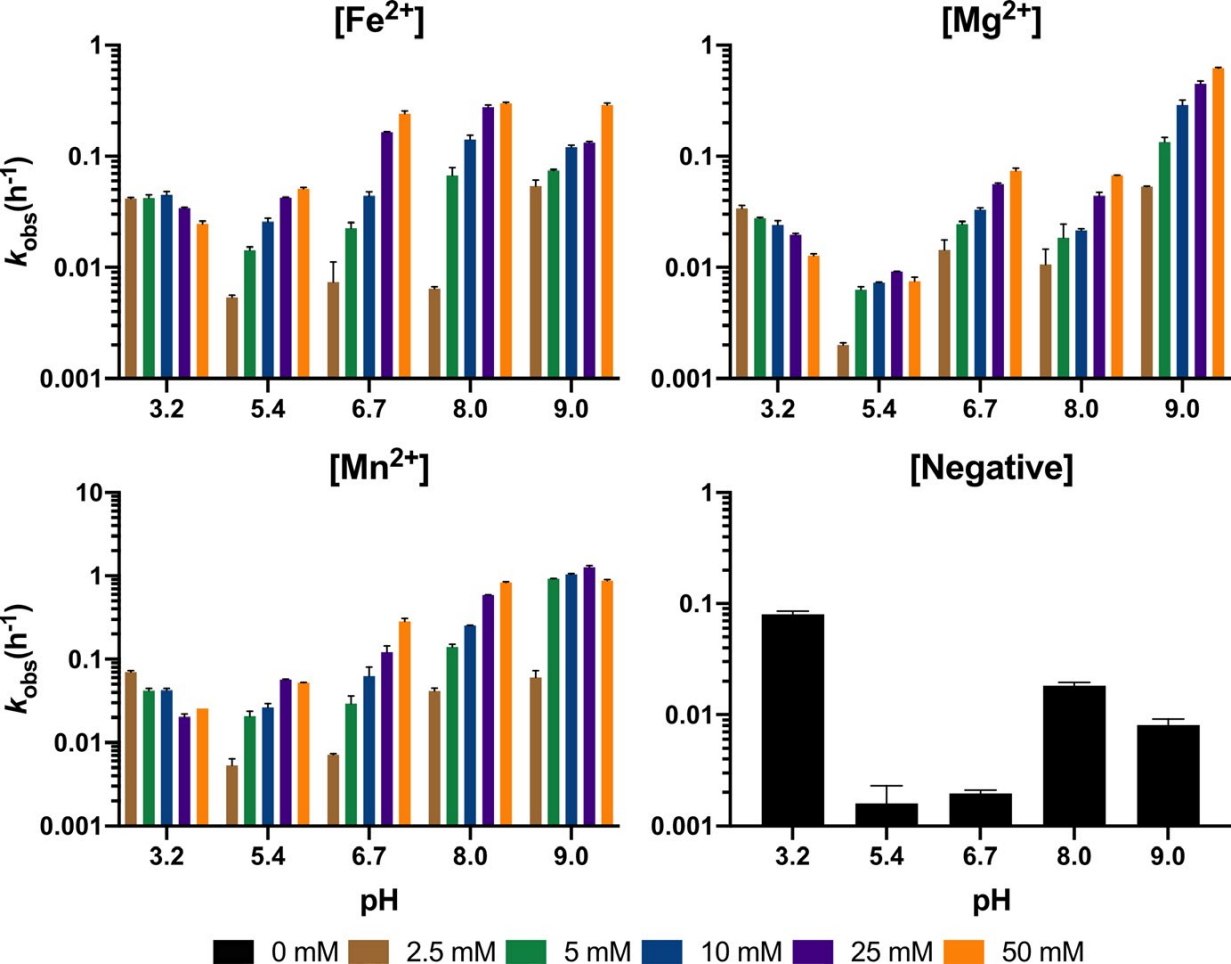
RNA degradation. Incubations of the RNA‐containing oligonucleotide at pH 3.2–9 and 0–50 mM of prebiotically relevant metal. Enhanced RNA hydrolysis occurs in nearly all pH conditions. At pH 3.2, increasing concentrations of metals decrease the rate of RNA degradation. Between pH 5.4 and 8, RNA incubations containing Mg2+ are generally more stable than those containing Fe2+ or Mn2+. At pH 9, we record the most rapid metal‐catalyzed hydrolysis rates in our experiments. Precipitates were observed for both Fe2+ and Mn2+ incubations likely resulting in artificially decreased catalysis rates relative to Mg2+. Error bars represent SEM (*n* = 2)

**Figure 6:**
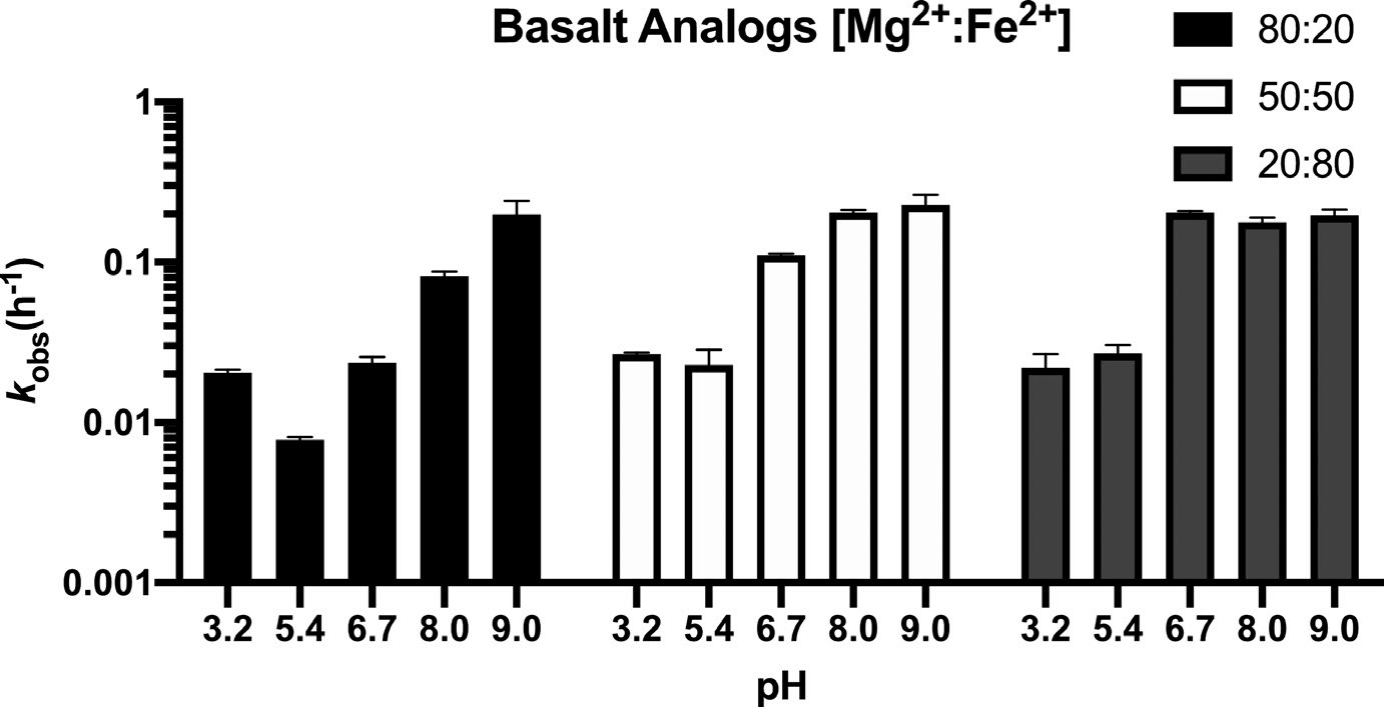
RNA degradation by metal mixtures. Results for the basalt analog solutions tend towards less overall cleavage in Mg2+‐rich solutions (e.g., forsteritic) over Fe2+‐rich solutions (e.g., fayalitic). Degradation at pH 9 for all basalt analogs is inferred to be primarily Mg2+‐catalyzed as Fe2+ was observed to precipitate out of solution. Error bars represent SEM (*n* = 2)

Our experimental results indicate that enhanced RNA cleavage occurs due to the presence of metals (i.e., metal‐catalyzed hydrolysis) in nearly all pH conditions (Figure 4, Table 1). However, at pH 3.2, increasing metal concentration decreases the rate of degradation (Figure 4). Catalysis at pH 3.2 is best mitigated by Mg^2+^ at 50 mM (*k*_obs_(h^‐1^) = 1.28 x 10^–2^) followed by Fe^2+^ at 50 mM (*k*_obs_(h^‐1^) = 2.47 x 10^–2^) then Mn^2+^ at 50 mM (*k*_obs_(h^‐1^) = 2.57 x 10^–2^) relative to the negative control (*k*_obs_(h^‐1^) = 8.06 x 10^–2^) (Figure 4, Table 1). Between pH 5.4 and 8, RNA incubations containing Mg^2+^ are generally more stable than those containing Fe^2+^ or Mn^2+^ (Figure 4, Table 1). This preference is most apparent in saturation curves fitted to a one‐site binding model which indicates a stability optimum at pH 5.4 where the maximum rate of RNA cleavage in the presence of Mg^2+^ (B_max_(h^‐1^) = 0.95 x 10^–2^) is notably lower than for Fe^2+^ (B_max_(h^‐1^) = 7.1 x 10^–2^) (Figure 5, Table 2). Trends for Mn^2+^ solutions are quantitatively similar to Fe^2+^ between pH 3.2 and 8 (Figure 4, Table 1). At pH 9, we record the most rapid metal‐catalyzed hydrolysis rates in our experiments. One‐site binding models show that Fe^2+^ (B_max_(h^‐1^) = 47.4 x 10^–2^) is more stable than Mg^2+^ (B_max_(h^‐1^) = 96.1 x 10^–2^) although we observed Fe^2+^ (and Mn^2+^) precipitate from solution (Figure 5, Table 2). Results for the basalt analog solutions tend toward less overall metal‐catalyzed hydrolysis in Mg^2+^‐rich solutions (e.g., forsteritic) over Fe^2+^‐rich solutions (e.g., fayalitic) (Figure 6).

**Table 1:** Pseudofirst‐order reaction kinetics k_obs_(h^‐1^) of RNA cleavage

Metal	pH 3.2			pH 5.4			pH 6.7			pH 8			pH 9		
	*k*_obs_ (h^−1^) (10^–2^)	SEM (10^–3^)	t_1/2_ (h)	*k*_obs_ (h^−1^) (10^–2^)	SEM (10^–3^)	t_1/2_ (h)	*k*_obs_ (h^−1^) (10^–2^)	SEM (10^–3^)	t_1/2_ (h)	*k*_obs_ (h^−1^) (10^–2^)	SEM (10^–3^)	t_1/2_ (h)	*k*_obs_ (h^−1^) (10^–2^)	SEM (10^–3^)	t_1/2_ (h)
[Fe^2+^]															
50 mM	2.47	1.40	28	5.07	1.70	14	24.1	14.5	3	30.0	6.10	2	28.9	13.2	2
25 mM	3.41	0.70	20	4.26	0.50	16	16.5	1.30	4	27.7	12.6	3	13.3	2.65	5
10 mM	4.49	3.05	15	2.58	1.85	27	4.40	3.65	16	14.1	13.8	5	12.1	5.50	6
5 mM	4.24	2.55	16	1.44	0.95	48	2.26	2.75	31	6.76	11.3	10	7.48	1.20	9
2.5 mM	4.17	1.10	17	0.54	0.25	130	0.74	3.80	94	0.65	0.25	107	5.40	7.00	13
[Mg^2+^]															
50 mM	1.28	0.50	54	0.75	0.70	92	7.42	3.75	9	6.76	0.30	10	62.3	11.3	1
25 mM	1.97	0.65	35	0.92	0.05	76	5.61	1.45	12	4.41	3.00	16	44.9	25.1	2
10 mM	2.43	2.05	29	0.73	0.10	95	3.31	1.30	21	2.16	0.70	32	29.0	31.2	2
5 mM	2.77	0.40	25	0.63	0.40	110	2.46	1.45	28	1.85	6.10	37	13.4	13.9	5
2.5 mM	3.40	2.15	20	0.20	0.10	347	1.44	3.20	48	1.06	4.10	65	5.32	0.60	13
[Mn^2+^]															
50 mM	2.57	0.05	27	5.22	0.35	13	28.6	25.5	2	82.7	20.7	1	87.5	29.9	1
25 mM	2.03	1.70	34	5.68	0.80	12	12.2	24.1	6	58.9	9.05	1	127	62.1	1
10 mM	4.24	2.00	16	2.64	2.90	26	6.29	18.0	11	25.4	3.10	3	104	27.9	1
5 mM	4.22	2.28	16	2.07	3.10	33	2.95	6.95	24	14.1	11.0	5	92.2	4.65	1
2.5 mM	7.03	3.10	10	0.53	1.10	131	0.72	0.25	97	4.20	3.00	17	6.04	12.9	11
Basalt Analogs [Mg^2+^:Fe^2+^]															
80:20	2.05	0.65	34	0.78	0.20	89	2.35	1.50	29	8.20	3.70	8	19.9	30.3	3
50:50	2.68	0.35	26	2.29	3.95	30	11.1	1.75	6	20.4	6.05	3	22.8	25.4	3
20:80	2.20	3.30	32	2.70	2.40	26	20.3	3.85	3	17.7	9.30	4	19.7	11.2	4
Negative Control															
0 mM	8.06	4.85	9	0.16	0.70	433	0.20	0.15	355	1.82	1.30	38	0.81	1.10	86

**Table 2:** One‐site binding model results for Fe^2+^ and Mg^2+^

Metal	pH	B_max_ (h^−1^) (10^–2^)	SEM (10^–3^)	K_d_	SEM
[Fe^2+^]	5.4	7.14	4.46	18.8	2.74
	6.7	90.9	469	134	89.7
	8	47.3	73.3	24.1	8.03
	9	47.4	147	38.7	22.1
[Mg^2+^]	5.4	0.95	0.99	3.97	1.59
	6.7	9.86	6.47	17.7	2.77
	8	11.7	21.1	37.8	12.7
	9	96.1	86.2	27.3	5.06

## 4 DISCUSSION

### 4.1 Prebiotically relevant metal catalysis

In aqueous solution, Mg^2+^, Fe^2+^, and Mn^2+^ form a hydrated hexa aquo species (Mg^2+^(H_2_O)_6_, p*K*_a_ = 11.4, Fe^2+^(H_2_O)_6_, p*K*_a_ = 9.6, and Mn^2+^(H_2_O)_6_, p*K*_a_ = 10.6) which polarize first shell water molecules in a tightly packed octahedral geometry (Jackson et al., 2015). Normally, these metal aquo complexes interact with secondary and tertiary RNA structures to neutralize the electrostatic repulsion of negatively charged phosphate groups brought into close proximity, or to increase local rigidity and join distal RNA structures by incorporating phosphate groups into their first coordination shell (Petrov et al., 2012). However, in our experiments, we sought to quantify the effect of metal‐catalyzed hydrolysis (i.e., RNA cleavage by transesterification), which is thought to be analogous to how certain ribozymes (e.g., the hammerhead self‐cleaving ribozyme) utilize metals to stabilize transition states and catalyze cleavage of the 5’,3’‐phosphodiester bond (Fedor, 2002; Hampel & Cowan, 1997; Johnson‐Buck et al., 2011). Namely, 1) acid/base interactions with water result in the activation of the ribose 2’‐hydroxyl nucleophile, and 2) first shell phosphate ligands draw electron density and expose phosphorous to nucleophilic attack. Attack of the 2’‐hydroxyl on the adjacent phosphate results in formation of a 2’‐3’ cyclic phosphate terminated oligonucleotide plus a second oligonucleotide product that begins with a 5’‐hydroxyl.

Our results appear to reproduce the enhanced RNA degradation rates expected to be associated with each metal's respective acid dissociation constant (p*K*_a_) in solution. Between pH 5.4 and 8, slower rates of metal‐catalyzed hydrolysis occur in the presence of Mg^2+^ (p*K*_a_ = 11.4) followed by Fe^2+^ (p*K*_a_ = 9.6) and then Mn^2+^ (p*K*_a_ = 10.6) (Figure 4, Table 1). Moreover, work on metal‐phosphorous complexes (i.e., M^2+^‐RNA clamp) has shown that because of low lying d orbitals, Fe^2+^ (0.11 e^‐^) has greater electron‐withdrawing power than Mg^2+^ (0.08 e^‐^) (Okafor et al., 2017) which likely compound the rate of RNA cleavage due to mechanism 2) described above (Hampel & Cowan, 1997). That is, Fe^2+^ is able to accept and retain electrons with greater affinity after its hydrated hexa aquo species loses a proton. At pH 9, it would appear that slower degradation rates occur in the presence of Fe^2+^ (B_max_(h^‐1^) = 47.4 x 10^–2^) rather than Mg^2+^ (B_max_(h^‐1^) = 96.1 x 10^–2^) contrary to our interpretation for pH 5.4 – 8 (Figure 5). Nonetheless, it is known that alkaline Fe^2+^ solutions will begin to form insoluble species such as Fe(OH)_2_ around pH ~ 9 (Gayer & Woontner, 1956) that might sequester Fe^2+^ from participating in catalysis. Precipitates were indeed observed for both Fe^2+^ and Mn^2+^ solutions albeit considerably more with Fe^2+^ at pH 9. This appears to corroborate interpretations that solubility properties are responsible for the observed differences between Mg^2+^ and Fe^2+^/Mn^2+^ under alkaline conditions (Jin et al., 2018). Results for pH 3.2 were unexpected as increasing concentrations of prebiotically relevant metals decreased the rate of degradation (Figure 4), and additional work is required to understand the precise preservation mechanism. However, researchers have proposed that perhaps metal ions can act as Lewis acids which stabilize the 2’‐hydroxyl group and prevent nucleophilic attack of phosphorous (Fedor, 2002). Results for the basalt analogs demonstrate greater degradation rates with increasing concentrations of Fe^2+^ relative to Mg^2+^ (Figure 6). This is best observed at pH 6.7 and 8 where 80:20 (20 mM Mg^2+^: 5 mM Fe^2+^) is mostly Mg^2+^‐dominated then transitions to Fe^2+^‐dominated at 20:80 (5 mM Mg^2+^: 20 mM Fe^2+^) (Table 1). RNA cleavage at pH 9 for all basalt analogs is inferred to be primarily Mg^2+^‐dominated as Fe^2+^ was observed to precipitate out of solution.

### 4.2 RNA on Mars—polymerization, stability, and redox environments

In order to fully evaluate the viability of an origin of life via the RNA world on Mars, we must consider synthesis in addition to stability. In other words, replication of RNA must be faster than degradation to effectively explore the fitness landscape. Work on non‐enzymatic RNA replication has demonstrated that metal catalysis actually increases the rate of polymerization by facilitating the deprotonation of the 3’‐hydroxyl group in 2‐methylimidazole nucleotides activating the 3’‐hydroxyl as a nucleophile (Li et al., 2017). Work by Jin et al. 2018 has also shown that Fe^2+^ facilitates RNA primer extension in solutions containing both template strands and monomers at a weakly acidic to neutral (pH ~ 7) optimum while Mg^2+^ is most effective at alkaline conditions (pH ~ 9). Conversely, while metal catalysis (e.g., degradation) is slower at acidic values considered in this study (Figure 4, Table 1) (e.g., Bernhardt & Tate, 2012), so is template copying chemistry (e.g., Jin et al., 2018). Altogether, pH catalysis optimums for Fe^2+^ and Mg^2+^ are particularly interesting as researchers have proposed that Fe^2+^ could have accordingly enabled an RNA world on the Earth's surface prior to the Great Oxidation Event (GOE) (e.g., Athavale et al., 2012). This is because some models predict reducing, circumneutral, and cool conditions throughout the Hadean (Charnay et al., 2017; Kadoya et al., 2020). Specifically, studies have demonstrated that Fe^2+^ may have preceded life's transition to Mg^2+^ due to its versatility in catalyzing polymerization (e.g., Jin et al., 2018), ribozyme folding (Athavale et al., 2012), translation (Petrov et al., 2015), and imparting novel catalytic activity (Guth‐Metzler et al., 2020; Hsiao et al., 2013; Okafor et al., 2017). It is therefore conceivable that Fe^2+^ could have had a comparable influence on the stability, polymerization, and function of RNA on early Mars and should accordingly be discussed below.

In situ observations of Mars suggest pH regimes ranging from acidic (pH 2 – 4) (e.g., Squyres & Knoll, 2005) to circumneutral (pH ~ 7) (e.g., Grotzinger et al. 2014) have existed on various locations under evolving redox conditions (Figure 2). Namely, pre‐ to early Noachian Mars is inferred to have been primarily reducing and neutral and progressively became oxidizing by the late Noachian resulting in pervasive acidic surface conditions (Figure 2) (Hurowitz et al., 2010; McLennan et al., 2019). Observations by MAVEN constrain the likely composition of an early atmosphere to primarily CO_2_‐dominated at ≥ 0.5 bars, which accordingly would not acidify surface environments below pH ~ 6.7 (Kua & Bada, 2011) pre‐ to early Noachian. Modeling of continental weathering of early Earth basalts additionally suggests that waters with high alkalinity would have stabilized pH between 6.6 and 7 under a ~ 1 bar CO_2_ atmosphere (Halevy & Bachan, 2017; Krissansen‐Totton et al., 2018). The global acidification of Mars is therefore presumed to be the result of increasing atmospheric O_2_ and/or photo‐oxidation of Fe^2+^ to Fe^3+^ as its atmosphere was lost (Hurowitz et al., 2010) which would consequently be highly detrimental to the prospects of an Fe^2+^‐mediated RNA world.

Our results indicate that a Mg^2+^‐rich basalt (e.g., McSween, 2002; Mustard et al., 2005) sourcing metals to a slightly acidic (pH 5.4) aqueous environment on Mars would have best supported long‐lived single‐stranded RNA polymers through the mid‐Noachian (Figures 2 and 5). Notwithstanding this observation, CO_2_ pressures (10 bar) required to sufficiently acidify surface waters are not supported by atmospheric models (e.g., Forget et al., 2013; Tian et al., 2010) as buffering from basaltic aquifers would neutralize pH as indicated above. Low pH values on Mars would have most likely been prevalent by the late Noachian (Figure 2) or perhaps locally at natural acid springs (e.g., Varekamp et al., 2009). Results from our experiments at pH 6.7 therefore represent the most accurate interpretation of potentially global conditions on Mars since the pre‐Noachian (Bibring et al., 2006). Assuming a cool (e.g., as indicated by atmospheric models), reducing, and UV‐shielded aqueous environment, this suggests an RNA world may have been relatively stable on the surface of Mars. A combination of Fe^2+^ and Mg^2+^ concentrations (as seen in basaltic porewaters) could have conceivably enabled novel catalytic activity and catalyzed polymerization while simultaneously tempering degradation rates due to differences in pH optimums (Figure 6). Depending on how early Mars became oxidizing, however, aqueous environments such as Meridiani Planum and Gusev Crater (pH 2–4) would accelerate RNA hydrolysis rates at low metal concentrations while simultaneously arresting polymerization at higher concentrations as observed at pH 3.2 (Figure 4). Most importantly, the oxidation of Fe^2+^ to Fe^3+^ produces a well‐known iron species that forms a strong complex with phosphate and leads to the precipitation of RNA (van Roode & Orgel, 1980). Increasingly oxidizing conditions on Mars near the mid‐late Noachian boundary would have likely limited Mg^2+^ as the primary catalyst for polymerization, folding, and hydrolysis outside of redox‐stratified environments like Gale Crater (Hurowitz et al., 2017) since the mid‐Noachian.

Overall, our results demonstrate that RNA stability depends on both metal concentration and pH. While concentrations up to 50 mM were tested here, lower metal concentrations ~ 1 mM are more geochemically plausible on Earth though estimates on Mars are not well constrained. Observations from anoxic crater lakes and perennially stratified ferruginous lakes on Earth show that ranges between 0 and 1.5 mM of dissolved Fe^2+^, Mn^2+^, or Mg^2+^ are reasonable for a basalt hosted basin (e.g., Bura‐Nakić et al., 2009; Busigny et al., 2014; Hongve, 1994; Kling et al., 1989). However, such low values are often at odds with proposed prebiotic chemistries which require 50 – 250 mM of metals for in situ RNA synthesis (e.g., 250 mM Fe^2+^ ‐ Patel et al., 2015) or replication (e.g., 50 – 200 mM Mg^2+^ ‐ Szostak, 2012). It is unclear whether such high concentrations of metals would be geochemically reasonable on early Mars; however, periods of wet‐dry cycling (e.g., playa environments) or endorheic lakes could conceivably facilitate required concentrations. Porewater chemistry simulations of the Gale Crater lacustrine phase vary greatly for Fe^2+^ (0.11–190 mM) and Mg^2+^ (0.64–210 mM) depending on modeled water‐rock interactions (Fukushi et al., 2019). Moreover, impact‐generated HCN in contact with surface waters precipitating ferrocyanide salts (e.g., CaK_2_[Fe(CN)_6_], MgNa_2_[Fe(CN)_6_]) has been proposed as a viable mechanism for concentrating metals over large timescales that are consistent with experimental synthesis of RNA, amino acid, and lipid precursors via cyanosulfidic chemistry (Patel et al., 2015; Sasselov et al., 2020; Toner & Catling, 2020).

Future work is required to further constrain the composition of theoretical Mars waters with respect to mechanisms that may have accumulated metals to prebiotically relevant concentrations (e.g., playas, brines, ferrocyanide salts). Furthermore, the study conducted here notably excluded the role of sulfates (e.g., Gendrin, 2005), which are found globally by the Late Noachian, on RNA stability. The work presented here highlights the importance of metals and pH derived from variable bedrock compositions and hypothetical atmospheric conditions on RNA stability. Additional studies will seek to include non‐enzymatic RNA extension, the effect of template and complement RNA strands, and additional environmental parameters such as UV flux. In summary, we believe the work presented here advances our understanding of how geochemical environments could have influenced the stability of a potential RNA world on Mars and on Earth.

## 5 CONCLUSIONS

Discoveries of structural and regulatory RNA molecules suggest that life as we know it may have emerged from an earlier RNA world (Bernhardt, 2012). However, due to global resurfacing and recycling (Marchi et al., 2014), it has been challenging to reconstruct the types of real‐world environments that may have existed on the Hadean Earth and hosted the origin(s) of life. We believe that Mars is the next best alternative to search for environments consistent with requirements imposed by the RNA world. In this study, we investigated the influence of bedrock composition (e.g., mafic‐ultramafic, iron‐rich, and magnesium‐rich) and subsequent weathering of prebiotically relevant metals (Fe^2+^, Mg^2+^, and Mn^2+^) on RNA stability. These metals have been demonstrated to catalyze hydrolysis (cleavage), folding, polymerization, and impart RNA with novel catalytic properties. In addition, we simultaneously adjusted pH to reflect the composition of hypothetical CO_2_‐dominated atmospheres in equilibrium with surface waters and waters at an acidic and alkaline vent. We determined that RNA stability depends on metal concentration and pH. Our results reproduce the enhanced RNA cleavage rates associated with each metal's respective acid dissociation constant (p*K*_a_) and an increase in metal concentration (Figure 4, Table 1). Degradation rates unexpectedly decreased with increasing metal concentration via an unknown preservation mechanism at pH 3.2 (Figure 4) though so do rates of polymerization (e.g., Jin et al., 2018). At pH 9, we encountered Fe^2+^ and Mn^2+^ precipitation which artificially decreased cleavage rates (Figure 4). We conclude that a Mg^2+^‐rich basalt sourcing metals to slightly acidic (pH 5.4) waters would therefore be the stability optimum (as determined here) for RNA on Mars. However, it is important to note RNA replication chemistry with Mg^2+^ as the metal cofactor requires mildly alkaline pH values in order to result in net accumulation (Jin et al. 2018). Geologic evidence and modeling of basalt weathering otherwise indicate that early Mars pore and (pre‐oxidizing) surface waters would have been near‐neutral pH ~ 7. Our experiments at pH 6.7 therefore represent the most accurate interpretation of potentially global conditions on Mars. Results from Fe^2+^ at this pH and prior work on iron catalysis suggest that while high cleavage rates decrease RNA stability, catalysis may result in net accumulation (e.g., in the presence of template strands and monomers). However, global oxidizing conditions (due to the lack of a dynamo) on the surface of Mars may have led to significant RNA instability due to the precipitation of RNA–Fe^3+^ complexes in Fe^2+^‐rich environments possibly as early as ~ 4.1 Gya. We therefore presume the non‐redox‐sensitive Mg^2+^ would have been the principal catalyst on Mars as hypothesized on Earth after the great oxidation event ~ 2.6 Gya (e.g., Athavale et al., 2012).

## AUTHOR DISCLOSURE STATEMENT

No competing financial interests exist.
